# Tuning the Electronic and Charge Transport Properties of Schiff Base Compounds by Electron Donor and/or Acceptor Groups

**DOI:** 10.3390/ma15238590

**Published:** 2022-12-02

**Authors:** Ahmad Irfan, Abdullah G. Al-Sehemi, Abul Kalam

**Affiliations:** 1 Department of Chemistry, College of Science, King Khalid University, P.O. Box 9004, Abha 61413, Saudi Arabia; 2 Research Center for Advanced Materials Science, College of Science, King Khalid University, P.O. Box 9004, Abha 61413, Saudi Arabia

**Keywords:** organic field-effect transistors, schiff base compounds, density functional theory, optoelectronic properties, charge transport

## Abstract

Organic semiconductors have gained substantial interest as active materials in electronic devices due to their advantages over conventional semiconductors. We first designed four Schiff base compounds, then the effect of electron donor/acceptor groups (methyl/nitro) was studied on the compounds’ electronic and transport nature. The absorption spectra (λ_abs_) were computed by time-dependent DFT at TD-B3LYP/6-31+G** level. The effect of different solvents (ethanol, DMF, DMSO, and acetone) was investigated on the λ_abs_. The substitution of the -NO_2_ group to the furan moiety at the 5th position in Compound **3** leads to a red-shift in the absorption spectrum. A smaller hole reorganization energy value in Compound **3** would be beneficial to get the hole’s intrinsic mobility. In contrast, a reduced-electron reorganization energy value of Compound 4 than hole may result in enhanced electron charge transfer capabilities. The reorganization energies of compounds **1** and **2** exposed balanced hole/electron transport probability. The optical, electronic, and charge transport properties at the molecular level indicate that Compound **3** is suitable for organic electronic device applications.

## 1. Introduction

Organic semiconductor materials (OSMs) have become very important due to their flexible, cheap, lightweight, and environmentally friendly nature [1,2,3]. Small OSMs are attracting interest because of their ease of synthesis, light weight, and inexpensive cost. The ability to interact with solvents and associated molecules for various modes of charge transport makes them beneficial for promising optoelectronic applications [4].

Developments in OSMs in organic field-effect transistors (OFETs), organic light-emitting diodes (OLEDs), photodiodes, and photovoltaics have persuaded the researchers to develop them into functional materials [5,6,7]. The OFETs with better performance may be achieved by the development of device fabrication approaches as well as by new materials [8]. Tuning excellent charge-transport properties in molecular systems is feasible by enhancing intermolecular interactions, resulting in rearranged optical and electronic structures. Thus, developing intermolecular interactions can be an efficient approach to enhance OFETs performance. To improve the intermolecular interactions of OSMs, heteroatoms like O, S, N and C=C or C≡C were substituted in the π-conjugated backbone. The existence of π-conjugation leads to the delocalization of electrons adjacent to the core and the prospect of superior charge carrier mobility [9,10,11,12]. OSMs are amalgamated by weak intermolecular interactions, like van der Waals, π-π, S–S, and C–H [13]. 

The OSM versatility delivers numerous exciting characteristics to be tuned. Since organic materials such as Schiff derivatives have potential in semiconductors, they have attracted great interest. Conjugated Schiff bases have thrilling properties and were used in numerous electronics [14,15], OFETs [16], and electrochromic devices [17]. The significant progress of electronic materials and devices in recent years has shown that the use of Schiff bases as electronic materials offers significant excellent advantages, and their facile synthetic process made them a potential alternative for electronic devices. The superior thermal strength, broader absorption, smaller band gap, and great electrical conductivity also ensures that Schiff bases will be used as future materials for organic electronics. The Schiff base compounds demonstrate intramolecular proton transfer and donor-acceptor combination.

Moreover, donor–π–bridge–acceptor and heteroatoms in these compounds govern the electronic transitions to make them appealing for semiconductors [17,18,19,20]. The specific feature of Schiff bases is their solvatochromic behavior. The π-conjugation leads to delocalization in these compounds resulting in superior mobility in OFETs. Moreover, donor and acceptor groups as substituents augment the electronic, solvatochromic, and optical properties [21].

**Figure 1:**
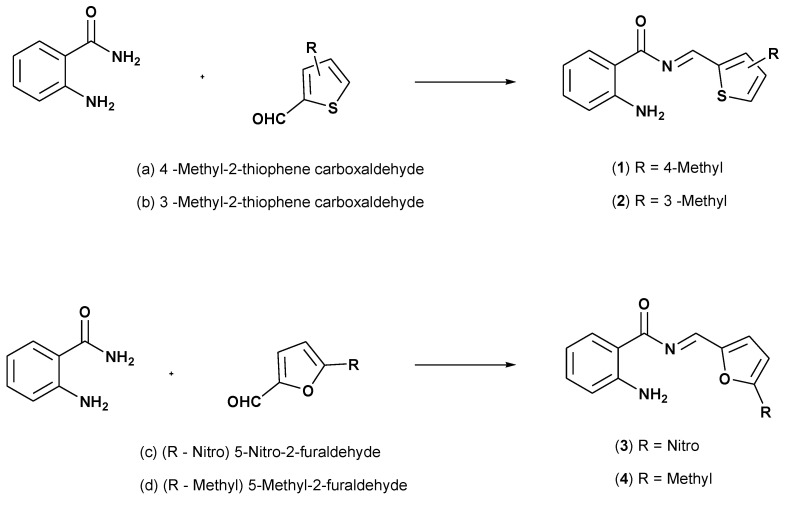
The structures of Schiff base compounds.

The effect of the electron-donating group (methyl), the electron-withdrawing group (nitro), and solvents with various polarities (ethanol, DMF, DMSO, and acetone) were examined on the absorption spectra for designed Schiff base compounds, see Figure 1. The donor and acceptor effect on the electronic and charge transport properties, e.g., frontier molecular orbitals, molecular electrostatic potential (MEP), and reorganization energy, is ascertained by DFT and time dependent DFT.

## 2. Computational Details

Quantum chemical approaches have proven efficient at exploring the structure–properties relationship. Computational investigations have played a substantial role in probing the properties of OSMs [22]. Computational methods are used to analyze materials’ properties in different fields [23]. It has been shown in previous studies that the density functional theory (DFT) is a rational choice for investigating the charge transport and optoelectronic properties of OSMS [24]. Moreover, quantum chemical calculations were utilized to ascertain the properties of thiophene-base compounds [25,26]. Optimization of Schiff base compounds in the ground state (S_0_) was accomplished by DFT [27,28,29,30] at the B3LYP/6-31+G** level, as the B3LYP functional demonstrated good results compared with reported data [31,32]. The electron affinity, ionization potential, [33] and reorganization energy (hole/electron) values were estimated at the B3LYP/6-31+G** level. Time-dependent DFT [34] was applied to calculate the absorption (λ_abs_) by TDDFT [35] the TD-B3LYP/6-31+G** level in gas and solvents (acetone, ethanol, DMSO, and DMF) using the Gaussian16 package [36].

## 3. Results and Discussion

### 3.1. Electronic Properties

The LUMO (*E_LUMO_*) and HOMO (*E_HOMO_*) and their energy gaps (*E_gap_*) are essential parameters to understand the optoelectronic and charge transport of compounds. Here, we have tabulated the *E_HOMO_*, *E_LUMO_*, and *E_gap_* of Schiff base compounds at S_0_ in Table 1. The electron-withdrawing group nitro instead of methyl was critical for adjusting the frontier molecular orbital (FMO) energy value in Comp**3**. Substitution of the nitro group reduces the *E_HOMO_* and *E_LUMO_* values of Comp**3** and decreases *E_gap_*, i.e., 2.07 eV.

**Figure 2:**
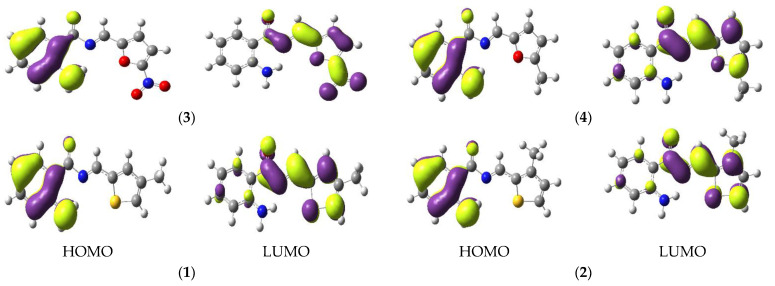
The ground state contours of FMOs (HOMOs and LUMOs) of Schiff derivatives (contour value = 0.035).

The charge densities distribution of FMOs are illustrated in Figure 2. The HOMO is delocalized on aminobenzene while LUMO is localized on thiophene/furan moiety in **1/2** and **3/4**. Moreover, LUMO can also be found at nitro group in **3** which is revealing an intra-molecular charge transfer (ICT) from H → L. 

The work functions (*ϕ*) of Ag (Al) are 4.74 (4.08 eV) [37]. The electron/hole injection energies (EIE/HIE) barrier was disclosed from Schiff base compounds to the Al/Ag electrode individually, which were assessed as (*E_LUMO_-ϕ*) and (*ϕ-E_HOMO_*), respectively. The estimated EIE barrier from Compounds **1**–**4** to Al are 1.40, 1.44, 0.27, and 1.53 eV, while HIE for **1**–**4** is 1.57, 1.54, 1.80, and 1.47 eV, respectively. The calculated EIE from **1**–**4** to Ag are 2.06, 2.02, 0.93, and 2.19 eV, respectively. The HIE for **1**–**4** are 0.91, 0.88, 1.14, and 0.81 eV. These findings uncovered that substituting the nitro group at the 5th position on the furan group in **3** would be promising to improve the electron injection. In contrast, the methyl group could complement the hole injection ability, see Table 2. 

### 3.2. Absorption Spectra

Absorption (λ_abs_) and the percent contribution of the transitions and oscillator strengths (*f*) involved by FMOs of Schiff base derivatives at TD-B3LYP/6-31+G** level in the gas phase and several solvents are tabulated in Table 3. The solvent effect has attracted much attention because many chemical processes occur in the solution phase. To study the impact of the solvent on absorption maxima, the gas phase studies have been made of molecules (as a control), and then the data were compared with the λ_abs_ and computed in a solvent such as ethanol, acetone, DMF, and DMSO. 

Here, we noticed that the first and second λ_abs_ peaks at 512 nm and 316 nm corresponded to H→ L (S_0_ → S_1_) and H→ L+1 for **1** in the gas phase, whereas the first and second λ_abs_ peaks were observed at 477 nm, corresponding to H→ L (S_0_ → S_1_) and 334 nm from H→ L+1 for **1** in ethanol. The effect of ethanol solvent in **1** was observed on λ_abs_ from the gas phase to ethanol, i.e., the first peak is 35 nm blue shifted while the second peak is 18 nm red shifted, respectively. The solvent polarity has no significant effect on λ_abs_ (see Table 3). In the gas phase, the substitution of the nitro group at the 5th-position of furan in **3** leads to a red shift, i.e., 233 nm in the first band and 94 nm in the second band as compared to **1**. The significant effect on the first peak was observed in λ_abs_ from the gas phase to ethanol in **3** which is being blue-shifted, i.e., 65 nm. The substitution of the nitro group at 5th-position of furan in **3** leads to a blue shift in λ_abs_ from the gas phase to acetone, DMF, and DMSO, i.e., 63, 63, and 67 nm for the first band, respectively. The effect of electron acceptor and electron donor moiety is mainly on the first λ_abs_ band, i.e., the substitution of the electron deactivating group (nitro) at the 5th-position of furan in **3** tune the λ_abs_ wavelength toward a shorter wavelength. 

All electronic transitions and corresponding oscillator strengths in the absorption spectra are π to π* type. A fascinating tendency in the oscillator strength was noticed for Schiff base compounds: the oscillator strengths for S_0_ → S_1_ are prodigiously larger in the solvent phase as compared to the gas phase. Furthermore, λ_abs_ indicates that **3** has a band in the visible region. According to the nature of the transition, the HOMO to LUMO transition was observed for the first excitation. The λ_abs_ wavelengths are listed in Table 3. All the compounds exhibit two absorption peaks related to the aminobenzene and the charge transfer associated with the thiophene/furan substituted moiety (Figure 2). The first bands may be attributed to a charge transfer-type transition (CT) of the π to π*, and from the HOMO located on aminobenzene to the LUMO on the thiophene-furan part. The lowest energy gaps are the 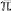 to 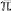
* for the **3** (2.07 eV), while the other Schiff based derivatives showed larger band gaps as established from the optical property values and the occurrence of UV values (see Table 3). These results clearly show that the interaction between the donor and the acceptor, either in an alternating manner or in a separate block in the molecule, performs a significant role in controlling the planarity and the photophysical properties.

### 3.3. Charge Transport Properties

Charge transports are sensitive to internal traps, as well as transport within *p*-type materials from low ionization potential levels, making the filling of deep traps less attractive. Likewise, materials of *n*-type benefit from a larger EA, where there are also few available traps. The hole injection from an electrode to a semiconductor HOMO is more effective when the electrode is closer, or even greater than a semiconductor IP. Similarly, for better electron injection, the larger EA values would be suitable. For the better stability of the device, it is certified that its charged and neutral states do not contribute to chemical reactions [38]. To preclude a thermodynamically efficient reaction (which contains oxygen and water), a neutral semiconductor is expected to require IP greater than 4.9 eV [38]. The shallow HOMO may diminish ambient O_2_ in the H_2_O existence to form OH^−^. The semiconductors having deep HOMO can receive holes that can oxidize the H_2_O in the atmosphere. Obstacles during such an undesirable reaction by chance lead to overpotentials that permit organic semiconductors to make redox gently so that they can unveil the stabilities. Electron transport is most affected by the reaction with air, and it is important to prevent the electron polaron from degrading the material. To achieve this, the LUMO has to be low to avert excited electrons to reduce the water-soluble O_2_ systems to O_2_^−^ [39] or H_2_O to OH^−^. These undesirable electrochemical processes can reduce charge transfer and set about irreversible changes within the semiconductor. Defining the exact amount of EA that needs to be skipped to prevent this redox reaction requires consideration of the maximum response power and device morphology. It has been suggested that high EA can put down oxidation reactions [40].

The values of the ionization potential (IPs) and the electron affinity (EAs) are among the most important factors of organic compounds for use in OFETs, xerography, and electroluminescence. These parameters have been interconnected to the amount of energy required to add or remove electrons and thus can be considered as molecular stability with respect to donating or receiving electrons to create an exciton. We describe the assessed IPs and EA for the Schiff base derivatives, see Table 4. Additionally, it entails that there was a positive correlation between the LUMO level and EAs. The 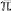-conjugated organic materials with an electronic charge motion are carried out by a hopping mechanism. The internal reorganization energy ($\lambda_{int}$), because of its structural variation from neutral to ionic states (cation and/or anion), is a vital parameter for the charge transfer in organic electronic materials, as it is one of the most important factors that influence the rate of the charge of hopping and considering the mobility of field-effect transistors. In order to have a high degree of mobility of materials, the $\lambda_{int}$ needs to be reduced. The $\lambda_{int}$ is a significant parameter for the estimation of the rate of charge transfer. Previously, it was exposed that the DFT can be a trustworthy way to imitate the experimental data [41,42,43,44]. The$\;\lambda_{int}$ and external polarization ($\lambda_{ext}$) are two components of total reorganization energy [45]. Here, $\lambda_{int}$ was projected for the hole ($\lambda_{hole}$) and the electron $\left(\lambda_{elec}\right)$. The $\lambda_{hole}$ was estimated by Equations (1) and (2) [46]:

*λ*_1_ = E^+^ (B) − E^+^ (B^+^) (1)

*λ*_2_ = E (B^+^) − E (B) (2)

where E^+^ (B), E^+^ (B^+^), E (B^+^), and E (B) are the energies of the cation at neutral optimized geometry, neutral at the cationic optimized geometry, optimized cation, and optimized neutral geometry, respectively. Correspondingly, $\lambda_{elec}$ was estimated by Equations (3) and (4):

*λ*_3_ = E^−^ (B) − E^−^ (B^−^) (3)

*λ*_4_ = E (B^−^) − E (B) (4)

where E^−^ (B), E^−^ (B^−^) and E (B^−^) are energies of the anion at neutral optimized geometry, neutral at anionic optimized geometry, and the anionic optimized geometry. 

The vertical and adiabatic EA and IP were computed by using Equations (5) and (6).

(5) $$\mathrm{Adiabatic}\;\{\begin{array}{c} IPa=E\left(B^{+}\right)-E\left(B\right)\\ EAa=E\left(B\right)-E\left(B^{-}\right) \end{array}$$

(6) $$\mathrm{Vertical}\;\;\;\;\;\{\begin{array}{c} IPv=E^{+}\left(B\right)-E\left(B\right)\\ \;EAv=E\left(B\right)-E^{-}\left(B\right) \end{array}$$

The computed $\;\lambda_{hole}$, $\lambda_{elec}\;$ and $\lambda_{int}$ of Schiff base derivatives at the level of B3LYP/6-31+G** are shown in Table 4. The smaller the electron reorganization energy value of the compound exposed that it would be suitable as an *n*-type semiconductor, while the smaller hole reorganization energy value of the compound uncovered that it would be appropriate as a *p*-type semiconductor; details can be found in reference 43. The $\lambda_{hole}$ and $\lambda_{elec}\;$ values for **1** and **2** reveal that these compounds might be good, having balanced hole and electron transfer mobility, and the ability to be used in *p* and *n*-type materials. The $\lambda_{hole}$ has been calculated to be much smaller than the $\lambda_{elec}\;$with the difference in the range of 0.151 eV for **3,** which reveals that this compound might be a suitable *p*-type contender. The $\lambda_{elec}\;$has been calculated to be smaller than the $\lambda_{hole}$ with the difference in the range of 0.045 eV for **4** which shows that this compound might be suitable as *n*-type contender. Hitherto, it was pointed out that lower $\lambda_{int}$ values can increase the charge transfer rate [43,47,48,49]. The substitution of the electron-deactivating group (nitro) at the 5th-position of the furan in **3** leads to a decrease in the polarization of the neutral to cation state which means that there are lower $\lambda_{hole}$ values compared to other compounds, suggesting that this compound could be a good choice for the hole transfer. It may turn out that the electron activating group (methyl) at the 5th-position of furan in **4**, leads to a decrease of the polarization of the neutral to anion which will result in a lower $\lambda_{elec}\;$value in comparison with other compounds, which suggests that this compound may be a good candidate for electron transport.

### 3.4. Molecular Electrostatic Potential

**Figure 3:**
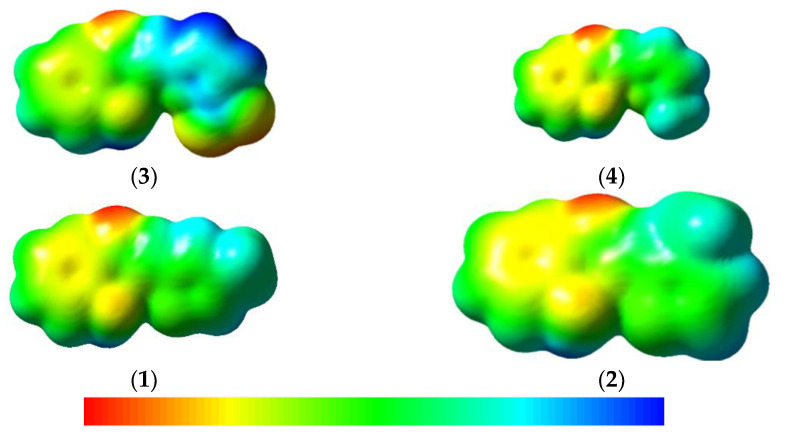
Molecular electrostatic potential views of Schiff base compounds.

Earlier works reported that diffraction methods would be supportive in determining molecular electrostatic potential (MEP) experimentally, and this can also be tested by simulation. The MEP points to the widespread electronic distribution across the board, which is a broad aspect of understanding and predicting the reactivity of different compounds. In Figure 3, the MEP map can be seen in the color scheme. Red represents regions with a high negative potential and blue indicates regions with high positive potential. High potential of negative regions is ideal for electrophilic attacks, however, high potential regions of positive prefer nucleophilic attacks. The MEP decreases with order blue > green > yellow > orange > red; red indicates the most repulsion while blue indicates the most attractive phase of the nucleophilic attack and vice versa.

From Figure 3, it was revealed that keto oxygen has negative potential in all the Schiff base compounds. The nitro group in **3** has also negative potential, while the -NH_2_ group has good positive potential in all the Schiff base derivatives. In **3**, the nitro group will have a negative potential. These results clearly indicate that in the event of a nucleophilic attack, the repulsion can be in potentially negative MEP areas. In addition, in the event of an electrophilic attack, the attraction may be to keto oxygen and the nitro group, while the greater the repulsion may be to the -NH_2_. The negative region indicates the photostability of the compounds.

## 4. Conclusions

Modification of chemical structures can improve the charge transfer, optical and electronic properties. These properties were tuned by substituting the electron donor and acceptor groups. These compounds were examined by B3LYP/6-31+G**, which allows a reliable estimate and analysis of the electronic structures of sulfur, nitrogen, and oxygen-containing organic molecules. The absorption wavelengths were estimated at the TD-B3LYP/6-31+G** level. The properties of donor–π–bridge–acceptor and acceptor–π–bridge–acceptor Schiff base compounds were explored. The substitution of -NO_2_ into the furan at the 5th-position in Compound **3** leads to red shift in the absorption spectra as compared to other molecules. The smaller hole reorganization energy value of Compound **3** would have an advantage to develop its hole intrinsic mobility. The smaller electron reorganization energy value for Compound **4** as compared to other counterparts would lead to better electron charge transfer ability. The results indicated that the designed Schiff base compounds would be good for organic electronic device applications, having competence as *n*-type (Compound **3**), *p*-type (Compound **4**), and balanced hole as well as electron transport materials (Compounds **1/2**).

## Figures and Tables

**Table 1:** The LUMO energies (*E_LUMO_*), HOMO energies (*E_HOMO_*), and energy gaps (*E_gap_*) (in eV) of Schiff base compounds at B3LYP/6-31+G** level.

Comp.	*E_HOMO_*	*E_LUMO_*	*E_g_*
**1**	−5.65	−2.68	2.97
**2**	−5.62	−2.64	2.98
**3**	−5.88	−3.81	2.07
**4**	−5.55	−2.55	3.00

**Table 2:** The hole/electron injection energy (EIE/HIE) barriers from of Schiff base compounds to Silver (Ag)/Aluminum (Al) electrodes were assessed at B3LYP/6-31+G** level.

Comp.	HIE (Ag)	EIE (Ag)	HIE (Al)	EIE (Al)
**1**	0.91	2.06	1.57	1.40
**2**	0.88	2.02	1.54	1.44
**3**	1.14	0,93	1.80	0.27
**4**	0.81	2.19	1.47	1.53

**Table 3:** The absorption wavelengths (λ_abs_, nm), oscillator strengths (*f*), %Contribution (%Con), and main transitions in the gas phase as well as in solvents (ethanol, acetone, DMF, and DMSO) of Schiff base compounds at TD-B3LYP/6-31+G** level.

Comp.	λ_abs_	*f*	Tran	%Con	λ_abs_	*f*	Tran	%Con
	Gas Phase				In Ethanol			
**1**	512	0.0432	H → L	71%	477	0.0721	H → L	71%
	316	0.2682	H → L+1	34%	334	0.4736	H → L+1	12%
**2**	510	0.0439	H → L	71%	474	0.0740	H → L	70%
	312	0.2970	H → L+1	47%	325	0.6117	H → L+1	60%
**3**	745	0.0259	H → L	71%	680	0.0343	H → L	70%
	410	0.0518	H → L+1	16%	334	0.5260	H → L+2	10%
**4**	502	0.0507	H → L	70%	468	0.0860	H → L	70%
	308	0.4157	H → L+1	37%	330	0.7663	H → L+1	12%
	230	0.0632	H → L+2	28%	240	0.0918	H → L+1	2%
Comp.	λ_abs_	*f*	Tran	%Con	λ_abs_	*f*	Tran	%Con
	In Acetone				In DMF			
**1**	478	0.0717	H → L	70%	477	0.0747	H → L	70%
	334	0.4744	H → L+1	12%	335	0.4839	H → L+1	12%
**2**	474	0.0736	H → L	70%	474	0.0769	H → L	70%
	325	0.6109	H → L+1	60%	326	0.6271	H → L+1	60%
**3**	682	0.0342	H → L	71%	682	0.0342	H → L	71%
	334	0.4860	H → L+2	9%	335	0.5838	H → L+2	9%
**4**	468	0.0855	H → L	70%	468	0.0893	H → L	70%
	330	0.7661	H → L+1	13%	332	0.7773	H → L+1	12%
	240	0.0918	H → L+1	2%	240	0.0921	H → L+2	3%
Comp.	λ_abs_	*f*	Tran	%Con				
	In DMSO							
**1**	477	0.0745	H → L	70%				
	335	0.4808	H → L+1	12%				
**2**	473	0.0766	H → L	70%				
	326	0.6244	H → L+1	60%				
**3**	678	0.0352	H → L	71%				
	335	0.5819	H → L+2	9%				
**4**	467	0.0891	H → L	70%				
	331	0.7750	H → L+1	12%				
	240	0.0926	H → L+2	3%				

**Table 4:** Vertical/adiabatic ionization potential (IP_v_/IP_a_), electron affinity (EA_v_/EA_a_), and hole/electron reorganization energies ($\;\lambda_{hole}$ /$\lambda_{elec}\;$ ) in eV of Schiff derivatives at B3LYP/6-31+G(d,p) level.

Comp.	IP_a_	EA_a_	IP_v_	EA_v_	$\lambda_{hole}$	$\lambda_{elec}$	Δλ
**1**	7.07	1.47	7.37	1.19	0.533	0.527	0.006
**2**	7.04	1.48	7.35	1.17	0.542	0.541	0.001
**3**	7.34	2.55	7.61	2.22	0.479	0.630	0.151
**4**	6.98	1.28	7.26	1.03	0.522	0.477	0.045

## Data Availability

Not applicable.
